# Novel downstream molecular targets of SIRT1 in melanoma: A quantitative proteomics approach

**DOI:** 10.18632/oncotarget.1898

**Published:** 2014-04-12

**Authors:** Chandra K. Singh, Jasmine George, Minakshi Nihal, Grzegorz Sabat, Raj Kumar, Nihal Ahmad

**Affiliations:** ^1^ Department of Dermatology, University of Wisconsin, Madison, WI; ^2^ Biotechnology Center, University of Wisconsin, Madison, WI; ^3^ The Commonwealth Medical College, Scranton, PA; ^4^ William S. Middleton VA Medical Center, Madison, WI

**Keywords:** Melanoma, SIRT1, Tenovin-1, Proteomics, BUB family proteins, shRNA

## Abstract

Melanoma is one of the most lethal forms of skin cancer and its incidence is continuing to rise in the United States. Therefore, novel mechanism and target-based strategies are needed for the management of this disease. SIRT1, a NAD(+)-dependent class III histone deacetylase, has been implicated in a variety of physiological processes and pathological conditions. We recently demonstrated that SIRT1 is upregulated in melanoma and its inhibition by a small-molecule, tenovin-1, inhibits cell proliferation and clonogenic survival of melanoma cells, possibly via activating p53. Here, we employed a gel free quantitative proteomics approach to identify the downstream effectors and targets of SIRT1 in melanoma. The human malignant melanoma, G361 cells were treated with tenovin-1 followed by protein extraction, in liquid trypsin digestion, and peptide analyses using nanoLC-MS/MS. A total of 1091 proteins were identified, of which 20 proteins showed significant differential expression with 95% confidence interval. These proteins were subjected to gene ontology and Ingenuity Pathway Analysis (IPA) to obtain the information regarding their biological and molecular functions. Real-Time qRT-PCR validation showed that five of these (PSAP, MYO1B, MOCOS, HIS1H4A and BUB3) were differentially expressed at mRNA levels. Based on their important role in cell cycle regulation, we selected to focus on BUB family proteins (BUB3, as well as BUB1 and BUBR1) for subsequent validation. The qRT-PCR and immunoblot analyses showed that tenovin-1 inhibition of SIRT1 resulted in a downregulation of BUB3, BUB1 and BUBR1 in multiple melanoma cell lines. Since tenovin-1 is an inhibitor of both SIRT1 and SIRT2, we employed lentivirus mediated silencing of SIRT1 and SIRT2 in G361 cells to determine if the observed effects on BUB family proteins are due to SIRT1- or SIRT2- inhibition. We found that only SIRT1 inhibition resulted in a decrease in BUB3, BUB1 and BUBR1. Our study identified the mitotic checkpoint regulator BUB family proteins as novel downstream targets of SIRT1. However, further validation is needed in appropriate models to confirm our findings and expand on our observations.

## INTRODUCTION

Melanoma is one of the most lethal forms of skin cancer. In the United States, 76,690 new cases of melanoma and 9,480 melanoma-related deaths were predicted for the year 2013 [1]. Epidemiological data suggests that age-adjusted annual incidences of melanoma have been on rise for the last 30 years [2]. The existing preventive or therapeutic approaches have not been able to effectively manage this deadly cancer and therefore, novel mechanism- and target- based approaches are needed for its management.

We have recently shown that the class III histone deacetylase (HDAC) SIRT1 is upregulated in human melanoma cells and tissues, and its small molecule inhibition by tenovin-1 causes anti-proliferative responses, which are mediated via activation of p53, in human melanoma cells [3, 4]. This is an interesting finding because the sirtuin (SIRT) family of NAD(+)-dependent protein deacetylases has been implicated in a wide range of biological processes, including genetic control of aging, regulating transcription, apoptosis, stress resistance and energy efficiency during low-calorie conditions [5-7]. SIRT proteins arbitrate post-translational alterations of the N-terminal tails of histone proteins, which bundle DNA into chromatin and play crucial roles in the regulation of gene expression [5]. Mammals possess seven SIRTs (SIRT1-7) that occupy different subcellular compartments such as the nucleus (SIRT1, -2, -6, -7), cytoplasm (SIRT1, -2) and the mitochondria (SIRT3, -4, -5), and exhibit different functions [8]. The role and functional significance of SIRTs in cancer development and progression is currently an intense area of research investigation [9-11]. SIRT1 has been shown to be upregulated in several cancers such as prostate cancer, cutaneous T-cell lymphoma, colorectal cancer and pancreatic cancer [11-15]. SIRT1 is also overexpressed in non-melanoma skin cancers, including squamous and basal cell carcinomas, actinic keratosis, and especially in Bowen's disease [16]. Further, the overexpression of SIRT1 has been linked to poor disease prognosis and survival in variety of cancers [17-19]. However, the role of SIRT1 is quite puzzling as there is an ongoing debate regarding its role as a tumor suppressor *versus* tumor promoter [20]. This makes it more important to study, in detail, the downstream targets of SIRT1.

Quantitative proteomics is an enthralling approach to acquire quantitative information regarding proteomes changes and offers promise in unveiling the complex molecular events in tumorigenesis, identification of cancer biomarkers and novel therapeutic targets [21, 22]. Gel free quantitative proteomics approaches are becoming more popular because of improved accuracy of nanoLC-MS/MS as well as advances in data analysis software which can expedite large scale data analysis. In this study, we employed gel-free quantitative proteomics to identify downstream targets of SIRT1 in melanoma.

## RESULTS

### Identification of SIRT1 downstream targets in melanoma by nanoLC-MS/MS analysis

**Figure 1:**
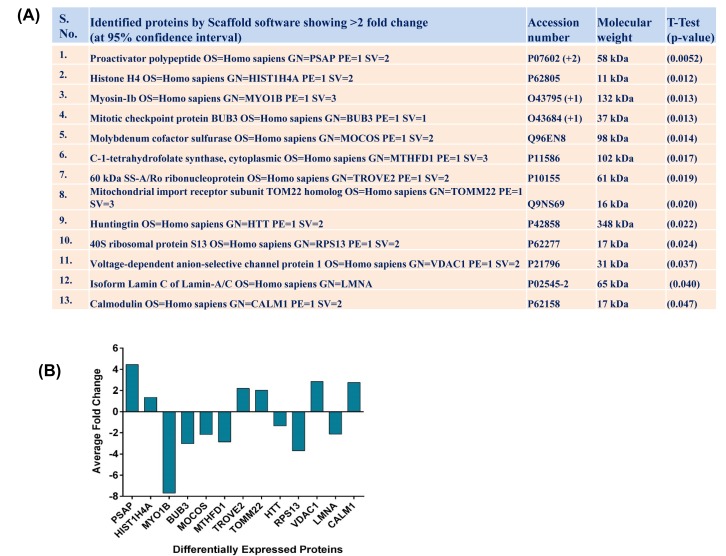
Effect of tenovin-1 on proteome changes in G361 cells Following nanoLC-MS/MS of vehicle control and tenovin-1 (25 μM) treated G361 cells, Scaffold software (version 3.6.3, Proteome Software Inc.) was used for protein annotations, identification and spectral based quantification. The top 13 proteins showing >2 fold change at 95% confidence interval are shown with their respective p-values in (A). Fold change of these differentially expressed proteins are plotted in (B). The data are representative of 3 biological replicates.

The goal of this study was to identify the possible downstream targets of SIRT1 involved in melanoma growth and progression. Using quantitative gel free proteomics, we analyzed the global proteome changes in response to SIRT1 inhibition by tenovin-1 in human melanoma G361 cells. NanoLC-MS/MS followed by Uniprot human database search revealed changes in a number of proteins (Supplementary Table 1). The results were highly reproducible as all three biological replicates produced similar results. The changes in proteins were accepted based on >95% probability with 2 unique peptides. In summary, total 1091 proteins were detected by Scaffold software in vehicle control (T0) and tenovin-1 (25 μM; T25) treated groups. Among these, 20 proteins showed 95% confidence interval (CI) with statistically significant differences (Supplementary Table 1). These significantly modulated proteins were subjected for Gene Ontology (GO) and Ingenuity Pathway Analysis (IPA). Of these 20 proteins, we selected 13 proteins (with >2 fold differences) for further validation by qRT-PCR analysis. These proteins were PSAP, HIST1H4A, MYO1B, BUB3, MOCOS, MTHFD1, TROVE2, TOMM22, HTT, RPS13, VDAC1, LMNA and CALM1. The details about these proteins including accession number, molecular weight and statistical analyses are provided in Fig. 1A. Interestingly, after tenovin-1 treatment, two of these proteins, HIST1H4A and TOMM22, appeared as new proteins, whereas, one protein HTT was found to disappear, following. The proteome profile of the fold change and direction of protein modulations are represented in Fig. 1B.

### Gene ontology analysis of proteome changes

**Figure 2:**
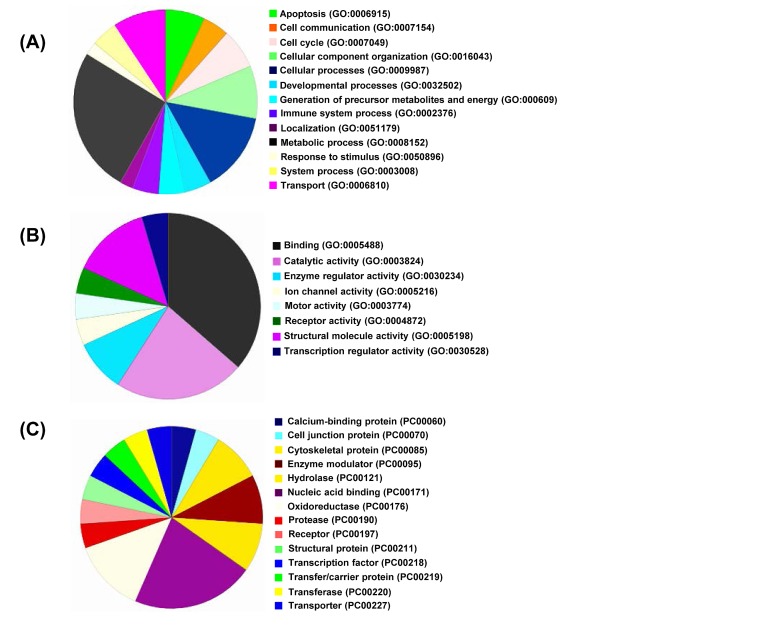
Gene Ontology analysis All the significantly modulated proteins identified in nanoLC-MS/MS (as detailed with bold blue color in Supplementary Table 1) were classified according to their GO descriptions and PANTHER classification systems and analyzed on the basis of Biological Processes (A), Molecular Functions (B), and Protein Class (C).

In order to obtain a global picture of proteome changes following tenovin-1 treatment, we subjected our proteomics data to GO database and PANTHER (Protein Analysis Through Evolutionary Relationships) classification to further categorize them according to their biological process, molecular functions and protein class (Fig. 2). As shown in Fig. 2A, the largest fraction of identified proteins belonged to cellular metabolism. This is not surprising as dysregulated cellular metabolism is a hallmark of cancer cells, and SIRT1 has been shown to affect metabolic processes [23]. Other major groups of proteins that showed changes included cellular component organization, biological organization, cell death, and cell cycle (Fig. 2A). Similarly, molecular function ontology identified binding, catalytic and structural molecule activity as the primary protein function; followed by other activities such as enzyme regulation, ion channel, motor, receptor, and transcription regulation (Fig. 2B). Further, protein class ontology indicated that the majority of proteins belonged to nucleic acid binding followed by oxidoreductase, enzyme modulator, hydrolase and cytoskeletal proteins (Fig. 2C). The other small percentages of modulated proteins fall in categories of calcium binding, cell junction, oxidoreductase, protease, receptor, structural, transcription, transfer/carrier and transporters.

### Pathway analysis for SIRT1 targets in melanoma by IPA software

**Figure 3:**
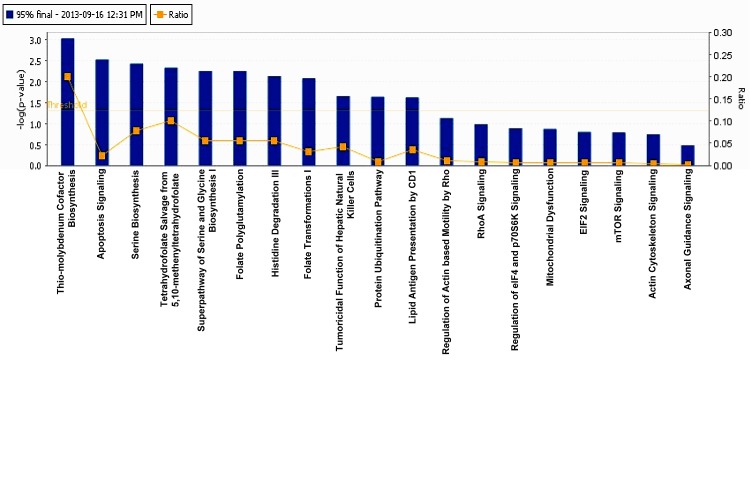
Ingenuity Pathway Analysis The proteins that showed significant change (95% confidence interval with statistical significance) were subjected for IPA analysis. The top 19 canonical pathways were identified as significantly altered upon SIRT1 inhibition by tenovin-1.

We used IPA software (trial version) to achieve molecular insight into the tenovin-1 mediated SIRT1 inhibition related proteome network in human melanoma cells. Proteins with 95% CI showing statistical significance among biological replicates were subjected for pathway analysis and network generation. Fisher's test was used to calculate the p-values associated with the canonical pathways. We identified 19 canonical pathways upon treatment of G361 melanoma cells with tenovin-1 (25 μM), among which thio-molybdenum biosynthesis and apoptosis signaling were the top hits (Fig. 3). Interestingly, dysregulation of apoptosis is a major hallmark of cancer cells, and it is not surprising to realize that tenovin-1 mediated SIRT1 inhibition may affect apoptosis signaling.

**Figure 4:**
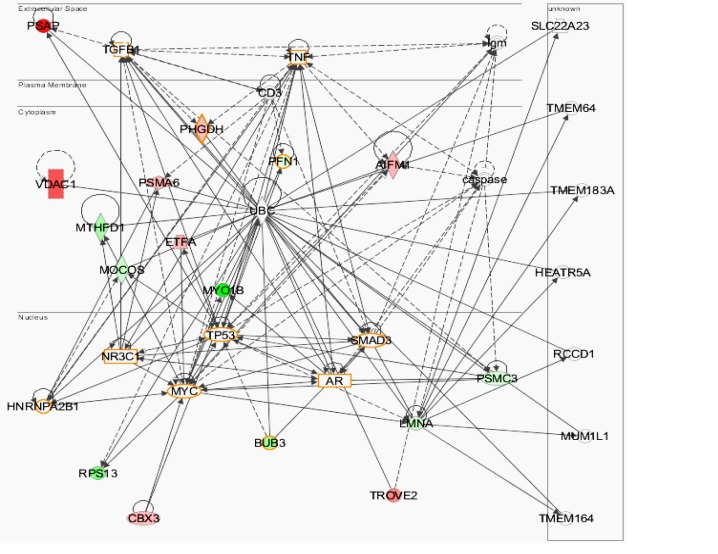
Protein-protein interaction by IPA analysis IPA was further used to analyze the protein-protein interactions and protein networks relevant to cancer. The solid lines denote a robust correlation with partner proteins, and dashed lines indicate statistically significant but less frequent correlations. The red color represents upregulated proteins whereas the downregulated proteins are shown in green. The un-colored nodes indicate additional proteins of this network that were not spotted by the proteomics analysis. The protein-protein interactions are indicated by arrows. The shape nodes denote the protein's function: enzymes (diamond); nuclear receptors (rectangle); transcription regulators (oval); cytokines (square); transporter (trapezoid); and others (circle).

Employing the IPA software, we further explored the proteins involved in cancer networks. These proteins are highlighted in different colors (Figure 4). Moreover, the protein-protein interaction analysis showed significant interactions among modulated proteins. These proteins formed a cluster where majority of proteins were connected with Ubiquitin C (UBC), a polyubiquitin precursor. This suggest that the majority of the modulated proteins are involved in ubiquitination via interacting with UBC as a process of post translational modification which might be affecting final action of the protein of interest (Fig. 4). Disrupted ubiquitination of proteins affects normal functioning of cells and leads to dysregulation of proteins that control cell growth and death. Frequent alteration of ubiquitination process has been noticed in cancer cells; which predominately affect the protein degradation, DNA repair, cell cycle regulation, cell proliferation, programmed cell death and regulation of other cell signaling pathways [24]. However, the modulation of SIRT1 and its association with UBC needs to be explored in melanoma and other cancers. Furthermore, protein network analysis by IPA highlights p53 as a central hub relating to MYC and other proteins from the connectivity map (Fig. 4). A recent study has suggested cooperation of SIRT1 with c-MYC in liver tumorigenesis [19]. Overall, this connectivity map suggests that p53 or p53-associated pathways are potential targets or effectors of the bioactivity of SIRT1 in melanoma. This observation is in agreement with the recently reported study by Lain and colleagues who showed tenovin-1 as an activator of p53, and work by inhibiting SIRT1 and SIRT2 [25]. Interestingly functional analysis revealed the connection of p53 with BUB3, a spindle checkpoint protein, which is frequently dysregulated in cancers [26-28].

### Real-Time qRT-PCR validation of identified downstream targets of SIRT1

Our next step was to explore the identified downstream targets of SIRT1 at the transcription level. For this purpose, we included two additional melanoma cell lines, A375 and Hs294T, in conjunction with G361. In addition, we used two concentrations of tenovin-1 (10 and 25 μM) for validation data. cDNA was isolated from all three cell lines treated with DMSO (T0), tenovin-1 10 μM (T10) and 25 μM (T25). A minimum of three biological replicates were used for final analysis presented in Table 1. Overall, we found that most of the modulated proteins identified in the proteomics analysis did not seem to follow the same trend at the transcription level, and were even different between the G361, A375 and Hs294T melanoma cells (Table 1). However, PSAP, MYO1B and BUB3 seem to follow the same trend as shown by proteomics analysis in all three melanoma cell lines. Therefore, the involvement of BUB1 and BUBR1, in addition to the mitotic regulator BUB3 was the subsequent focus of our study.

**Table 1:** Real-Time qRT-PCR validation

Target Gene	G361			Hs294T			A375		
	T0	T10	T25	T0	T10	T25	T0	T10	T25
VDAC1	1.00 + 0.01	0.66 + 0.06b	0.69 + 0.08b	1.00 + 0.02	0.69 + 0.04c	0.86 + 0.04a	1.01 + 0.08	1.27 + 0.13	1.60 + 0.05b
TROVE2	1.00 + 0.02	0.66 + 0.06	0.96 + 0.18	1.00 + 0.06	1.05 + 0.21	1.10 + 0.28	1.01 + 0.07	1.58 + 0.29	1.92 + 0.29a
PSAP	1.01 + 0.06	1.20 + 0.14	1.53 + 0.11a	1.01 + 0.09	1.19 + 0.12	1.84 + 0.16b	1.01 + 0.08	2.44 + 0.57	3.07 + 0.61a
MYO1B	1.00 + 0.01	0.52 + 0.03d	0.35 + 0.04d	1.00 + 0.04	0.49 + 0.03d	0.36 + 0.02d	1.00 + 0.04	0.93 + 0.05	0.66 + 0.02c
CALM1	1.00 + 0.01	0.74 + 0.06	0.75 + 0.12	1.00 + 0.04	0.53 + 0.01d	0.60 + 0.02d	1.02 + 0.11	1.33 + 0.08a	1.74 + 0.03c
TOMM22	1.00 + 0.02	0.60 + 0.01	0.89 + 0.21	1.01 + 0.08	0.95 + 0.11	0.74 + 0.09	1.06 + 0.21	1.83 + 0.24a	1.46 + 0.11
MTHFD1	1.01 + 0.08	0.42 + 0.04	0.73 + 0.22	1.01 + 0.10	0.72 + 0.10	0.59 + 0.04a	1.01 + 0.07	0.78 + 0.19	0.88 + 0.04
HTT	1.00 + 0.01	0.80 + 0.02	1.18 + 0.20	1.00 + 0.03	0.87 + 0.10	0.89 + 0.03	1.07 + 0.21	2.04 + 0.23a	2.49 + 0.22b
LMNA	1.00 + 0.01	0.79 + 0.04	1.15 + 0.26	1.00 + 0.04	0.86 + 0.05	0.79 + 0.03b	1.00 + 0.02	1.14 + 0.05	1.23 + 0.07a
HIST1H4A	1.00 + 0.04	1.35 + 0.17	0.37 + 0.13b	1.01 + 0.07	0.27 + 0.09a	0.47 + 0.20	1.00 + 0.02	0.33 + 0.05d	0.25 + 0.05d
RPS13	1.00 + 0.03	0.60 + 0.01a	0.87 + 0.15	1.00 + 0.01	0.64 + 0.03d	0.62 + 0.01d	1.02 + 0.11	1.29 + 0.17	1.21 + 0.13
MOCOS	1.00 + 0.01	1.03 + 0.11	2.11 + 0.41a	1.00 + 0.01	1.39 + 0.07c	1.72 + 0.05d	1.00 + 0.06	2.63 + 0.23b	4.25 + 0.33d
BUB3	1.00 + 0.02	0.51 + 0.02d	0.36 + 0.06d	1.00 + 0.02	0.37 + 0.02d	0.36 + 0.01d	1.00 + 0.03	0.80 + 0.06a	0.78 + 0.03a

Real-Time qRT-PCR analysis were performed to validate the protein changes at mRNA levels in melanoma cells. cDNA synthesis and PCR assays were carried out as detailed in ‘Materials and Methods’. Data are represented as mean value + standard errors of minimum three biological replicates. Statistical significance is represented as: a = P<0.05, b = P<0.01, c = P<0.001 and d = P<0.0001.

### BUB family proteins as novel downstream targets of SIRT1

**Figure 5:**
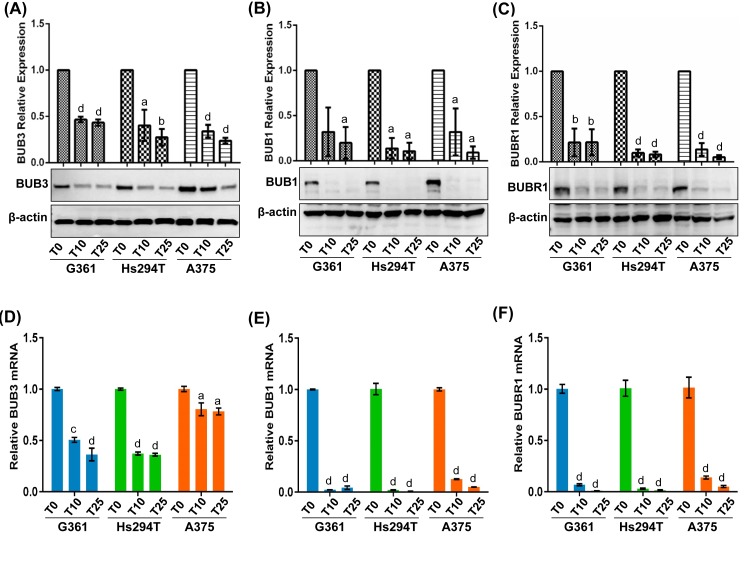
Immunoblot validation of BUB family proteins Immunoblot analyses of vehicle control (T0) and 10 and 25 μM tenovin-1 (T10 and T25) treated samples were performed as detailed in ‘Materials and Methods’. Representative blots showing expression of BUB3 (A), BUB1 (B) and BUBR1 (C) are shown. Quantitative fold change was calculated with respect to vehicle control on the basis of pixel density for each band (normalized to β-actin), as calculated by Kodak Image Station 4000MM software. Protein data were further confirmed at transcription level by qRT-PCR assay as detailed in ‘Materials and Methods’. The mRNA expression level of BUB3 (D), BUB1 (E), and BUBR1 (F) in melanoma cells G361, A375 and Hs294T are presented. Data are represented as mean value + standard errors of minimum three biological replicates. Statistical significance is represented as: a = P<0.05, b = P<0.01, c = P<0.001 and d = P<0.0001.

BUB family proteins play major roles in the process of the mitotic-spindle checkpoint which makes crucial decisions in the cell cycle [29, 30]. Therefore, as the next step, we analyzed the effect of tenovin-1 on protein levels of BUB3, BUB1 and BUBR1 in all three melanoma cell lines. Control and treated cell lysates were analyzed by immunoblot analyses. As shown in Fig. 5, tenovin-1 treatment was found to result in a significant decrease in BUB3, BUB1 and BUBR1 proteins in melanoma cells. The expression patterns of these proteins were found to be consistent even at the mRNA level in all three melanoma cell lines tested (Fig. 5).

**Figure 6:**
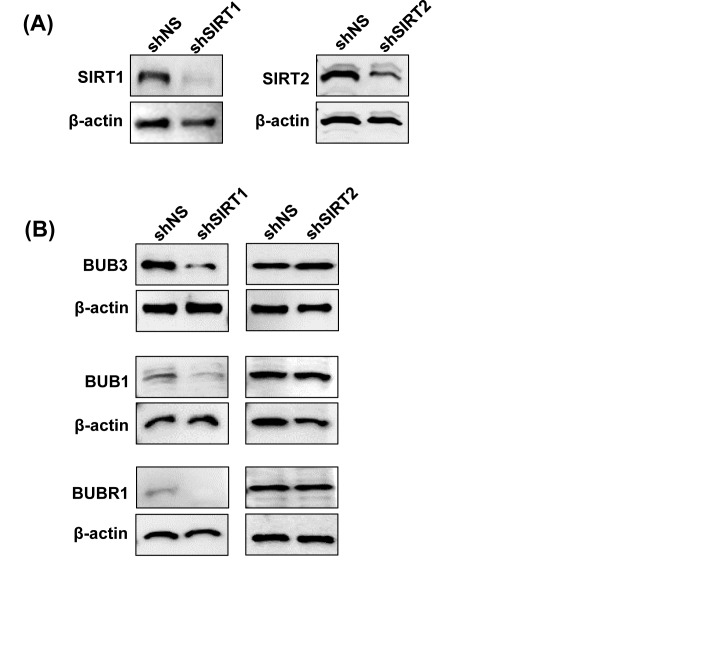
Effect of SIRT1 and SIRT2 knockdown on BUB family proteins Following SIRT1 and SIRT2 knockdown, immunoblot analyses were performed as described in ‘Materials and Methods’. Knockdown was confirmed by probing with SIRT1 or SIRT2 antibodies (A), and effect on BUB3, BUB1 and BUBR1 protein levels were assessed following SIRT1 and SIRT2 knockdown (B). Equal loading was confirmed by re-probing the blots with β-actin.

As tenovin-1 is not a specific inhibitor of SIRT1 and is known to inhibit SIRT2 as well, we were interested to find out if the observed changes in BUB family proteins were due to SIRT1-inhibition, SIRT2-inhibition or a combination of both. To accomplish this, we knocked down SIRT1 and SIRT2 separately in G361 cells using lentiviral shRNA approach. As shown in Fig. 6A, a genetic knock down of SIRT1 and SIRT2 resulted in a nearly complete inhibition of their respective expression. Interestingly, we found that only SIRT1 knockdown resulted in a decrease in BUB3, BUB1 and BUBR1 proteins, whereas, SIRT2 knockdown did not affect these proteins (Fig. 6B). This suggests that under the experimental conditions employed in our study, SIRT1 modulates BUB signaling in melanoma cells.

## DISCUSSION

The major objective of this study was to identify the downstream targets of the class III HDAC SIRT1 in melanoma cells using gel-free proteomics. This is important because overexpression of SIRT1 has been reported in a variety of cancers, including non-melanoma skin cancers [11-16]. We have recently demonstrated that 1) SIRT1 is upregulated in human melanoma, and 2) tenovin-1 mediated inhibition of SIRT1 resulted in an anti-proliferative response in melanoma cells, which is mediated by increase in p53 activity [3, 4]. However, SIRT1 has been shown to modulate a vast range of cellular activities [23, 31, 32]. Therefore, it is important to try to identify additional downstream targets of SIRT1. Such information could be useful in designing novel strategies for the management of melanoma and possibly other cancers. Gel free based quantitative proteomics approach is a useful technique for identifying targeted signaling pathways especially when changes in protein expression can be related to physiological and biochemical parameters that respond to the treatment of interest [21, 22]. Employing the nanoLC-MS/MS gel free approach, 1091 proteins were found to be modulated by tenovin-1 inhibition of SIRT1 in human melanoma cells G361. Of these, 20 proteins had 95% confidence interval with a statistical relevance. The analysis of modulated proteins by GO and PANTHER classification showed that tenovin-1 affects proteins that regulate cellular metabolic pathways, protein binding and catalytic activity, and nucleic acid binding, and oxidoreductase protein class. Our findings are interesting because on the way to tumorigenesis, cells undergo metabolic reprogramming to fulfill their energy requirement. Further, SIRT1 has been shown to be involved in a large variety of metabolic processes [23].

**Figure 7:**
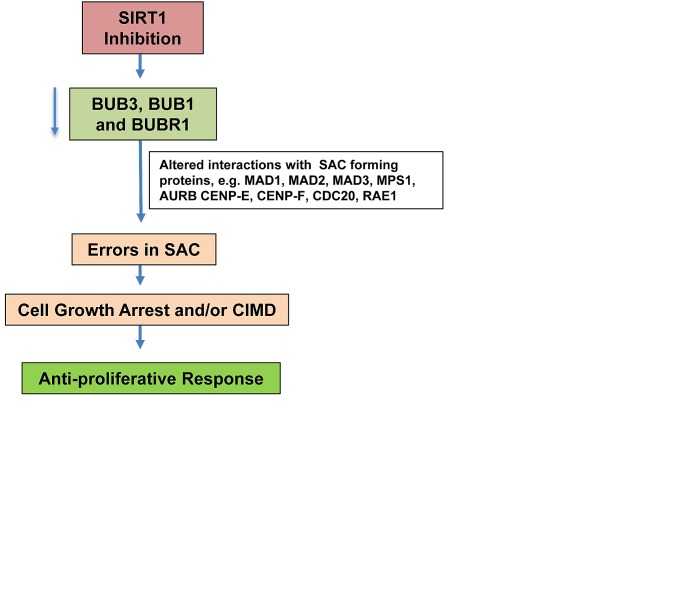
Schematic representation of possible mechanism of SIRT1 inhibition mediated antiproliferative responses in human melanoma cells

In order to interpret the differentially expressed proteins, predominant canonical pathways and interaction networks were generated by IPA, which is based on regularly updated database consisting interactions between proteins selected from scientific literature. The most significant canonical pathways (based on p-value) were thio-molybdenum biosynthesis and apoptosis pathways. Furthermore, as evident from the connectivity map, p53 was shown to act as a central hub related to most of the modulated proteins. p53 plays a large role in the cellular response to stresses and DNA damage largely through transcriptional repression or activation of gene of interest. p53 remains undetectable under normal conditions due to tight regulation by MDM2-mediated p53 degradation [33]. During cellular stress, p53 becomes stabilized and induces cell cycle arrest and DNA damage repair, or promotes cellular senescence or apoptosis [33]. p53 is extensively studied in cancer research, and p53 function is often blunted by genetic mutations in cancer cells, thereby leading the cancer cells to bypass cell cycle arrest and apoptosis processes [34]. Our protein network data showed the interactions of p53 with a majority of the modulated proteins, as well as with BUB3. Since mitotic checkpoint proteins play a critical role in regulating the cell cycle and proliferation, we chose to further determine the effect of SIRT1 inhibition on BUB3 and other BUB family proteins (BUB1 and BUBR1) involved in mitotic checkpoint regulation, especially at the spindle assembly checkpoint (SAC), which ensures proper chromosome segregation, anaphase onset and chromosomal attachment to the spindle [35]. BUB family proteins (BUB3, BUB1, BUBR1) together with several other interacting proteins form the SAC and prevent the action of Anaphase Promoting Complex (APC), and thereby stop early anaphase entry and mitotic exit [29, 30]. At unattached kinetochores, BUBR1, BUB3 and MAD2 interact with CDC20 and inhibits the formation of active APCCdc20 [36]. The BUB1/BUB3 complex plays a role in the inhibition of APC when spindle-assembly checkpoint is activated and inhibits the ubiquitin ligase activity of APC/C by phosphorylating its activator CDC20 [36]. This makes BUB family proteins (BUB3, BUB1, BUBR1) important regulators of SAC formation and signaling. We have found that SIRT1 inhibition leads to the depletion of BUB proteins (BUB3, BUB1, BUBR1) that might result in a dysfunctional SAC, and thereby cell arrest or, possible induction of Caspase-Independent Mitotic Death (CIMD) (Fig. 7). Kitagawa and Niikura have reported that cells choose to undergo CIMD as a result of BUB1 depletion to avoid chromosome mis-segregation caused by reduced SAC functioning [37]. Our data suggest the involvement of BUB family proteins in the anti-proliferative response of SIRT1 inhibition in melanoma cells. The clinical relevance of BUB proteins is yet to be revealed in melanoma. However, BUB1 and BUBR1 inhibition has been shown to enhance radiation sensitivity in pediatric glioblastoma cells [38]. Additionally, overexpression of BUB3, BUB1 and BUBR1 proteins has been reported in gastric cancer, clear cell kidney carcinomas and breast cancer [26-28, 39]. Hu and colleagues recently reported that BUBR1 is overexpressed in esophageal squamous cell carcinoma that contributes to resistance to paclitaxel (an anti-microtubule drug); and its knockdown was found to restore sensitivity to paclitaxel [40]. Defective cell cycle checkpoints are considered fascinating targets for anti-cancer therapies, especially due to the resulting disruption in the mitotic spindle apparatus, which leads to cell cycle arrest and subsequently to the induction of tumor cell death [35].

Taken together, our study has identified novel insights into SIRT1 signaling in melanoma by connecting this important protein deacetylase with spindle assembly check point proteins BUB3, BUB1 and BUBR1. However, further validation is needed in appropriate models to confirm our findings and expand on our observations.

## MATERIALS AND METHODS

### Cell culture and treatment

G361, Hs294T and A375 human melanoma cells and human embryonic kidney HEK293T cells were obtained from American Type Culture Collection (ATCC, USA). G361 cells were maintained in McCoy's 5a medium (Corning Cellgro, USA), and Hs294T, A375, HEK293T in Dulbecco's Modified Eagle's Medium (Corning Cellgro, USA) with 10% FBS (Sigma, USA) at standard cell culture conditions (37°C, 5% CO2 in humidified incubator). Cells were grown to approximately 50% confluence, and treated for 48 h with 10 and/or 25 μM tenovin-1 (Cayman Chemical, USA) dissolved in DMSO. DMSO alone treated cells served as control.

### Preparation of protein lysates

Following treatments, cells were lysed in 1X RIPA buffer (EMD Millipore Corp., USA) containing protease inhibitor cocktail (Thermo Fisher Scientific Inc., USA) and 1 mM PMSF (Amresco LLC, USA) on ice for 30 min. The cell lysates were spun down at 10,000 × g for 10 min at 4°C. The supernatant was saved and the protein concentration was established using the Pierce BCA Protein Assay Kit (Thermo Fisher Scientific Inc., USA). The lysates were stored at -80°C until use for nanoLC-MS/MS and immunoblotting.

### Enzymatic “In Liquid” digestion

“In Liquid” digestion and mass spectrometric analysis was done at the Mass Spectrometry Facility (Biotechnology Center, University of Wisconsin-Madison). Briefly, 200 μg of methanol:chloroform extracted crude protein lysates of G361 treated with DMSO as vehicle alone (T0) or 25 μM tenovin-1 (T25) were re-solubilized and denatured with 8 M urea in 50 mM NH_4_HCO_3_ (pH 8.5) for 10 min. The samples were then reduced by the addition of 25 mM DTT in 25 mM NH_4_HCO_3_ (pH 8.5) also containing 1 mM Tris-HCl (pH 7.5) to reduce carbamylation and incubated for 15 min at 50°C. This was followed by the addition of 55 mM iodoacetamide (IAA) and incubated in the dark at room temperature for 15 min; reaction was quenched by adding 8 μl of 25 mM DTT. Protease digestion was performed with the addition of 100 ng/μl Trypsin Gold (Promega Corp., USA) in 25 mM NH4HCO_3_. The digestion was conducted for 1 h at 42°C then an additional 20 μl of trypsin solution was added and the reaction was allowed to proceed overnight at 37°C. The reaction was subsequently terminated by acidification with 2.5% trifluoroacetic acid and 8 μl (~13 μg) was loaded for nanoLC-MS/MS analysis.

### NanoLC-MS/MS

Peptides were scrutinized by nanoLC-MS/MS using the Agilent 1100 nanoflow system (Agilent Technologies, USA) connected to a hybrid linear ion trap-orbitrap mass spectrometer (LTQ-Orbitrap XL, Thermo Fisher Scientific Inc., USA) equipped with a nanoelectrospray ion source. HPLC was carry out using an in-house fabricated 15-cm C18 column filled with Jupiter 4 μm C12 particles (Phenomenex Inc., USA) and laser pulled tip (P-2000, Sutter Instrument) using 360 μm x 75 μm fused silica tubing. Sample loading (8 μl) and desalting were done at 10 μL/min using a trapping column in line with the autosampler (Zorbax 300SB-C18, 5 μM, 5x0.3mm, Agilent Technologies, USA). Peptide elution used solvents comprised of 0.1% formic acid in water (solvent A) and 0.1% formic acid, 95% acetonitrile in water (solvent B). The gradient comprised of a 20 min loading and desalting time with column equilibration at 1% solvent B, an rise to 40% B over 195 min, ramp to 60% B over 20 min, rise to 100% B in 5 min and hold for 3 min. The column was then re-equilibrated at 1% solvent B for 30 min. The flow rate for peptide elution and re-equilibration was 200 nl/min. The LTQ-Orbitrap was set to obtain MS/MS spectra in data-dependent mode as follows: MS survey scans from m/z 300 to 2000 were accumulated in centroid mode at 100,000 resolving power. MS/MS spectra were saved on the 5 most-abundant signals in each survey scan. Dynamic exclusion was used to amplify dynamic range and increase peptide identifications. This feature omitted precursors up to 0.55 m/z below and 1.05 m/z above earlier selected precursors. Precursors remained on the exclusion list for 40 sec. Singly-charged ions and ions for which the charge state could not be allotted were excluded from consideration for MS/MS.

### Database search and protein identification

Raw MS/MS data was searched against Uniprot human amino acid sequence database formatted to include reverse sequences and common contaminants [70,130 protein entries] using in-house Mascot search engine 2.2.07 [Matrix Science, USA] with fixed Cysteine carbamidomethylation and variable Methionine oxidation with Asparagine and Glutamine deamidation. Peptide mass tolerance was fixed at 20 ppm and fragment mass at 0.8 Da. Protein annotations, significance of identification and spectral based quantification was done with help of Scaffold software (version 3.6.3, Proteome Software Inc., USA). Peptide identifications were recognized if they could be ascertained at greater than 95.0% probability as restricted by the Peptide Prophet algorithm [41]. Protein probabilities were assigned by the Protein Prophet algorithm, and only those proteins were accepted showing >95.0% probability with at least 2 identified peptides. [42]. Proteins with similar peptides that could not be distinguished based on MS/MS analysis alone were clustered to fulfill the principles of parsimony.

### Pathway and protein-protein interaction analysis

The modulated proteins identified in nanoLC-MS/MS were classified according to their Gene Ontology descriptions using information from the GO database and PANTHER (http://www.pantherdb.org/) classification systems. The predicted protein-protein interaction networks and canonical pathways were generated by Ingenuity Pathway Analysis Software (IPA trial version, Ingenuity Systems, www.ingenuity.com) (Qiagen, USA) using inputs of gene identifiers, log2 fold-changes and p-values between two-group comparisons.

### Quantitative real time RT-PCR (qRT-PCR) analysis

Total RNA was isolated from the control, 10 and 25 μM tenovin-1 treated G361, A375 and Hs294T cells with the QIAshreder and RNeasy Mini Kit (Qiagen, USA) and first strand cDNA was transcribed with random primers, dNTPs and M-MLV reverse transcriptase (Promega, USA). qRT-PCR was performed using the StepOnePlus Real-Time PCR system (Life Technologies Corp.) and SYBR Premix Ex Taq II (TaKaRa, USA) with first strand cDNA, forward and reverse primers. Primers for VDAC1, TROVE2, PSAP, MYO1B, TOMM22, MTHFD1, HTT, HIST1H4A, RPS13, MOCOS, BUB1 and GAPDH were selected from the PrimerBank database [43]. Primer sequence for BUB3, BUBR1, LMNA and CALM1 were selected from respective references [39, 40, 44, 45]. All the primer sequences used in this study are listed in Supplementary Table 2. The PCR program was set as: initial denaturation step (95°C for 30 s) followed by DNA amplification (95°C for 3 s followed by 61°C for 30 s) for 40 cycle. Melt curve analysis was performed to ensure the specificity of target amplicon. GAPDH was used as an endogenous control and ΔΔCT algorithm was used for relative quantification of target amplicons.

### Lentiviral production and generation of stable SIRT1 and SIRT2-knockdown G361 cells

Virus particles were produced by transfection of HEK293T cells with control vector shRNA (pLKO.1, SHC002), SIRT1 shRNA (TRCN0000018983, Clone ID: NM_012238.3-1958s1c1) and SIRT2 shRNA (TRCN0000040218, Clone ID: NM_012237.2-1739s1c1) (Sigma-Aldrich, USA) using the CaPO_4_ method as described previously [46]. The virus-containing medium was harvested at 72 h following transfection, and filtered using a 0.45 μm filter (Millipore, USA). For target cell transduction, G361 cells were passaged to 40% confluence in a six well plate, and viral media was added to the cells with 8 μg/ml polybrene four times over two days. After 72 h of transduction, viral media was replaced with selection media containing 2 mg/mL puromycin. The cells were cultured for 3 weeks in 2 μg/ml puromycin for stable clone selection. Transduction of G361 cells with the shRNA-expression plasmids resulted in the cell lines G361-shSIRT1 and G361-shSIRT2, respectively. A nonsense encoding G361-shNS served as a control. SIRT1 and SIRT2 specific knockdown was confirmed in stable cells using immunoblot analysis.

### Immunoblotting

For immunoblot analysis, 40 μg protein was subjected to SDS-PAGE, transferred onto a nitrocellulose membrane and blocked with 5% non-fat dry milk in 1X TBST buffer. The membrane was probed with SIRT1, SIRT2 (Santa Cruz Biotechnology, USA), BUB3, BUB1 or BUBR1 (Abcam, USA) primary antibodies followed by appropriate HRP-conjugated secondary antibodies, and analyzed by chemiluminescent detection system (Thermo Fisher Scientific Inc., USA). The blots were re-probed with β–actin (Cell Signaling, USA), a reference protein to demonstrate equal loading of protein samples.

### Statistical analysis

The LC-MS/MS data from three biological replicates were analyzed using Scaffold_3.6.1 (Proteome Software Inc.). Student's t-test was applied as appropriate way to tell if the abundances are different between the treated (T25) and DMSO control (T0) groups. The results of a t-test are reported as the probability (p-value) that this distance between means could occur by chance. The qRT-PCR data were analyzed using StepOne Software v2.2 RQ Study (Life Technologies Corp.) and exported as RQmax and RQmin (2^-ΔΔCt +/- ΔΔCt SD^) which represent relative quantity for gene of interest. For the analysis of immunoblots, densitometry was accomplished using Kodak Image Station 4000MM (Carestream Health, Inc., USA) and normalized to β-actin loading control. Further, statistical analyses on biological replicates of qRT-PCR and immunoblot data were performed with GraphPad Prism 5 software (GraphPad Software, Inc., USA) using one-way analysis of variance (ANOVA) followed by Dunnett's multiple comparison test.

## SUPPLEMENTARY TABLES


